# Cancer mortality in small areas around nuclear facilities in England and Wales.

**DOI:** 10.1038/bjc.1984.261

**Published:** 1984-12

**Authors:** J. A. Baron

## Abstract

Cancer mortality trends were examined for the small areas around fourteen nuclear and five non-nuclear facilities in England and Wales. Using routine OPCS mortality data, standardized mortality ratios (SMRs) for these areas were computed for selected causes of death. Changes in the SMRs were then sought by comparing the SMRs for the five years before the facility opened with the period 10 (in some cases 15) years after start-up, and by computing the weighted regression of the SMRs on calendar year. These analyses indicate no overall pattern of increasing cancer SMRs around nuclear facilities.


*Br. J. Cancer* (1984), **50**, 815–824

### Cancer mortality in small areas around nuclear facilities in England and Wales

J.A. Baron

*Norris Cotton Cancer Center and Department of Medicine, Dartmouth Medical School, Hanover, New Hampshire 03756, USA.*

**Summary** Cancer mortality trends were examined for the small areas around fourteen nuclear and five non-nuclear facilities in England and Wales. Using routine OPCS mortality data, standardized mortality ratios (SMRs) for these areas were computed for selected causes of death. Changes in the SMRs were then sought by (1) comparing the SMRs for the five years before the facility opened with the period 10 (in some cases 15) years after start-up, and (2) by computing the weighted regression of the SMRs on calendar year. These analyses indicate no overall pattern of increasing cancer SMRs around nuclear facilities.

The continuing public debate regarding the health effects of nuclear facilities has included discussion of increased cancer risk in the populations surrounding them. It is generally thought that the likely risks from the known radioactive emissions are negligible (Taylor & Webb, 1978; Comptroller General, 1981). However, estimation of the size of this possible radiation hazard is hampered by controversy regarding dose-reponse effects at low doses (Advisory Committee on the Biological Effects of Ionizing Radiation 1980; Land, 1980) and uncertainty regarding the actual dosage delivered by releases of known amounts of radiation (Commission of the European Communities, 1979). These difficulties make attractive an epidemiologic approach.

There have, in fact, been several studies of disease patterns in areas surrounding nuclear installations (Tokuhata & Smith, 1981; Patrick, 1977; Geary *et al.*, 1979; Enstrom, 1983). Almost all showed no significant effects, but many were limited by investigation of small populations, by a lack of control (non-exposed) areas, or by a lack of continued surveillance over time. Most focused on sites in the United States.

This report presents an analysis of cancer mortality in small areas around fourteen nuclear and five non-nuclear facilities in England and Wales. Where appropriate, aggregation of data into groups of sites has allowed a study population which is both substantial in size and close geographically to at least one facility. Small area mortality data routinely collected by the Office of Population Censuses and Surveys (OPCS), have permitted an analysis of trends over extended periods of time.

As there is substantial geographic variation in cancer mortality in Britain (Gardner *et al.*, 1983), relatively high or low cancer mortality in any small area may well be unrelated to the presence of a nearby facility. Therefore this study focused on *changes* in cancer mortality after the nuclear sites became operative.

### Methods

Estimates of radioactive discharges from nuclear facilities in England and Wales were used to identify those reporting the largest radiation releases in the late 1970s (Department of the Environment, 1980). Using ordnance survey maps, the location of each of these was identified and any pre-1974 local government authority with a majority of its area within a 5-mile radius was designated as "associated". If there was no such area, an increasing radius was used until at least one local authority area was included whose closest boundary was not separated from the facility by a major geographical division (such as an ocean bay) or by a distance of greater than two miles. Any area wholly surrounded by an associated area was also considered associated. All geographic units used were as defined prior to the 1974 local government reorganization (Population Statistics Division, Office of Population Censuses and Surveys, 1979), since the time span of this study was largely prior to that date. The year when a site became operative was taken to be the year in which significant nuclear activity began (when a research reactor became functional, when a power station went into commercial operation, etc.) (Gowing, 1974; United Kingdom Atomic Energy Authority, 1979; Nuclear News, 1983). The same procedure was followed for five oil, coal, or gas-burning electric power stations. These were chosen such that their non-urban setting and dates of start-up (R.W. Prior,

Received 10 May 1984; accepted 19 September 1984.

© The Macmillan Press Ltd., 1984

1982, Personal communication) were similar to those of the nuclear facilities. None was within thirty miles of a major nuclear facility, though all were within standard regions (Registrar General, 1934 *et seq.*) that did contain a nuclear facility. Of the ten plants that met these criteria, five were arbitrarily selected to include sites in various parts of the country.

For each local authority area, yearly numbers of observed deaths by sex for selected causes were obtained from OPCS routine small area mortality records (series SD25 and SD30) (Davies & Chilvers, 1980). The causes of death tabulated varied depending on year. During 1939–1944, there were no small area records at all. For the preceding years, available and relevant were numbers of deaths from all causes, and from all malignancies combined. During 1945 to 1949, counts of deaths due to cancer of the stomach, breast, and uterus (all sites) were added. Beginning in 1950, also available were tabulations of deaths due to leukaemia (all types combined) and cancer of the lung. In the late 1960s and 1970s, many more causes were tabulated. Because of the limited time span with this information available, these were not considered here. For each site, data were collected for the period beginning five years prior to the start-up and extending through 1979, the last year for which data was available when this study began.

Expected numbers of deaths were obtained by multiplying England and Wales death rates for a given year by the estimated age/sex-specific populations of the local authority areas. The age groups used in this calculation were 0–4 years, then successive decades to 74, and 75 years old or greater. These death rates were calculated by dividing the yearly number of deaths in England and Wales for a given age interval by its midyear population (Registrar General, 1934 and seq.). For the years up to 1973, the age- and sex-specific population estimates for a local authority area were calculated by multiplying the yearly Registrar General's total population estimates (Registrar General, 1934 and seq.) by age/sex proportions obtained from linear interpolation between censal estimates of these proportions.

For the years 1974 and 1979, the local government reorganization necessitated additional computation, since yearly population estimates for pre-1974 areas were not published by OPCS after 1974. However, OPCS has computed 1981 census populations for pre-1974 towns and cities (Office of Population Censuses and Surveys, 1982). This allows computation of total population estimates for the years 1974–1979 for urban districts, municipal boroughs, and county boroughs, through interpolation between the 1973 and 1981 estimates. For rural districts, total population estimates were made for 1974–1979 by assuming that over this period the old areas formed constant population proportions of the reorganized ones (Office of Population Censuses and Surveys, 1975), for which OPCS did compute yearly population estimates (Office of Population Censuses and Surveys, 1974 *et seq.)*. Because of a lack of age-specific 1981 census data for interpolation, the age/sex structure of the local areas for 1971 was assumed throughout the years 1971–1979.

For each year and cause of death, the observed and expected numbers of deaths were separately summed over the local authority areas associated with each facility. These observed and expected totals were then separately summed over years to obtain totals pertaining to time intervals of interest. Standardized mortality ratios (SMRs) were then calculated for each cause by dividing the observed number of deaths by the expected and multiplying this observed to expected ratio by 100.

For each facility, changes were sought in the relative mortality in its associated areas. Firstly, the SMRs for a "baseline" time interval were compared with those for the aggregated period fifteen or more years after the start-up date. (The relatively recent start-up of the nuclear electricity stations necessitated a ten-year intervening interval for those facilities.) These intervals were chosen to allow for a latent period of any effects. The "baseline" was defined as the 5-years just prior to start-up if the appropriate small area death records were available; if not, the earliest possible 5-year period was used. However, Little Barford and Amersham began operation so early that no meaningful baseline could be assigned for leukaemia or lung cancer. Statistical testing of the differences between SMRs was performed using one-degree of freedom chi-square tests (Levine *et al.*, 1980). Confidence limits around the SMRs can be computed easily from the data given in Tables II and III (Bailar & Ederer, 1964).

As a second assessment of change in relative mortality, the weighted regression of the SMRs on year was performed using data from the start-up year or later and the expected values as weights (Armitage, 1971). The regression slopes can be interpreted as the average annual percent change for an SMR of 100. They address the overall trend in the SMRs in the years after a facility opened, while the before/after comparison contrasts the SMR in years immediately prior to start-up with that from a much later period. The two techniques thus focus on somewhat different time spans in addition to employing different methodologies. Slopes were presented only if there was at least a minimal linear pattern in SMRs as indicated by a corresponding regression sum of squares greater than 4% of the total sum of squares (Armitage, 1971). (For unweighted regression, this would have meant requiring a correlation at least 0.2 in magnitude.) No statistically significant slopes were eliminated from consideration by this procedure. These regression calculations were also applied to data for the aggregate of all rural districts in England and Wales during 1950 to 1973, years for which the relevant population data is available from OPCS (Registrar General, 1934 *et seq.*).

Because the facilities within each of the nuclear electric and conventional electric groups are similar with regard to fuels and emissions (Dept. of the Environment, 1980), pooled estimates of trends were calculated for each of these two groups. This was done by fitting a common slope to the relevant set of regression lines while allowing for possibly different intercepts (Armitage, 1971). Testing for heterogeneity of the individual slopes in the group was also performed (Armitage, 1971).

Small area age-specific mortality information for the causes of death noted above were available for the years 1963 and later. This permits calculations of SMRs for childhood cancer (0–14 years old) near the nuclear electricity facilities over virtually their entire period of operation. Because of small numbers of deaths, only the "before/after" comparison was used in analyzing these data. Two 8-year time spans were contrasted: 1963–1970 and 1972–1979. This resulted in a comparison of the "early" versus the "late" years after a facility opened. A similar analysis was not possible for the other groups of plants because of their earlier start-up dates.

The cause of death designations utilized were the ICD codes as employed by OPCS. Six ICD revisions were used during the time span considered (ICD versions 4–9). There were also minor changes in the disease groupings used in the SD25 and SD30 series at OPCS. From 1950 through 1967, cancers of the trachea, bronchus, and lung were tabulated together as a unit, while in succeeding years only cancers of the lung and bronchus were included. In 1979, all three were again considered together. These are all discussed here as "cancer of the lung." Similarly, between 1945 and 1949 cancer of the duodenum was grouped with cancer of the stomach, which was tabulated alone in later years. These are all referred to here as "cancer of the stomach."

### Results

The nuclear and non-nuclear facilities included in the analysis are listed in Table I, along with their associated local authority areas, start-up times, and 1970 populations. The nuclear facilities are largely in rural areas; most are in the south of England and Wales. A majority began operation after 1960; a few became active in the 1940s. Oldbury itself was not considered further, since its associated local authority areas were also associated with Berkeley, which had an earlier start-up date. Wylfa was also dropped from further consideration because its recent start-up permitted only a few years of observation.

The before/after comparisons are presented in Tables II and III. As might be expected, there was considerable systematic variation in the SMRs between establishments and between causes of death, while differences from year to year were more random. Around the conventional electricity generating facilities the SMRs for the later years showed no consistent relationship to baseline. As expected in a large data set, several of the comparisons were statistically significant, showing increases or decreases in no apparent pattern. There were no statistically significant increases in the SMRs for all malignancies or leukaemia around any of these facilities, though the later SMRs were statistically higher than baseline for all causes near Richborough, and all causes and cancer of the stomach around South Denes.

For the nuclear electricity facilities, the before/after comparisons were similar, in that no overall pattern emerged. The later SMRs were consistently higher than baseline around Dungeness, but consistently lower around Bradwell. (Around both stations only the all cause contrast was statistically significant.) There were significant increases in all cause SMRs around Sizewell, and in uterine cancer and leukaemia around Trawsfynydd. There were significant decreases for cancer of the breast around Berkeley, and all causes around Trawsfynydd.

The data for childhood cancer around the nuclear electric facilities (Table III) likewise showed no consistent trend. All cause relative childhood mortality decreased around all the facilities except Trawsfynydd and Sizewell, where increases were not statistically significant. The increase in the SMRs for all childhood malignancies around Trawsfynydd was also not statistically significant, and not of sufficient magnitude to explain the rise for all causes combined. There were relatively few observed deaths from childhood malignancies near any facility, and no statistically significant differences for all malignancies or leukaemia between the early and late periods. Trawsfynydd had much higher later leukaemia SMRs than baseline, but both ratios were based on very small numbers and the difference was not statistically significant.

Among the remaining nuclear facilities, Amersham and Windscale had all-ages SMRs that

**Table I** Study sites, start-up dates and associated pre-1974 local authority areas.

| | *Start-up date* | *1970 population* | *Association local authority areas* |
|---|---|---|---|
| *Conventional electricity generating facilities* | | | |
| Little Barford | 1941 | 14,630 | St. Neot's UD |
| Fleetwood | 1955 | 58,920 | Fleetwood MB, Thornton-Cleveleys UD, Preesall UD |
| Portishead | 1955 | 8,740 | Portishead UD |
| Richborough | 1962 | 112,750 | Margate MB, Ramsgate MB, Sandwich MB, Broadstairs and St. Peters UD |
| South Denes | 1957 | 50,180 | Gt Yarmouth CB |
| *Nuclear electricity generating facilities* | | | |
| Bradwell | 1962 | 37,340 | Maldon RD, Maldon MB, West Mersea MB |
| Berkeley | 1962 | 78,050 | Dursley RD, Lydney RD, Thornbury RD |
| Hinkley | 1965 | 67,210 | Bridgewater RD, Bridgewater MB, Burnham-on-Sea MB, Watchet MB |
| Dungeness | 1965 | 4,380 | Lydd MB |
| Wylfa | 1971 | 13,900 | Twrcelyn RD, Almwch UD |
| Transfynydd | 1965 | 6,930 | Deudraeth RD |
| Sizewell | 1966 | 8,450 | Leiston-cum-Sizewell MB, Aldeburgh MB |
| Oldbury | 1968 | 57,420 | Lydney RD, Thornbury RD |
| *Other nuclear facilities* | | | |
| Amersham | 1940 | 106,470 | Amersham RD, Chesham UD, Beaconsfield UD, Chorleywood UD |
| Aldermaston | 1952 | 39,130 | Bradfield RD |
| Springfields | 1948 | 146,300 | Fylde RD, Kirkham UD, Fulwood UD, Preston CB |
| Capenhurst | 1953 | 58,180 | Ellesmere Port MB |
| Harwell | 1947 | 71,310 | Wallingford RD, Wantage RD, Abingdon MB, Wantage MB |
| Winfrith | 1960 | 29,280 | Wareham and Purbeck RD, Wareham MB |
| Windscale | 1950 | 46,900 | Millom RD, Ennerdale RD |

RD = rural district.
UD = urban district.
MB = municipal borough.
CB = county borough.

were generally lower in the later period than during baseline (Table II). These decreases were statistically significant only for all causes around Windscale and cancer of the stomach around Amersham. Conversely, around Harwell the later SMRs were generally higher than baseline. These increases achieved statistical significance for all causes and all malignancies combined. although the rise for laekaemia was the largest. Springfields also had non-significantly higher later leukaemia SMRs in contrast to lower all cause SMRs in the late period.

The regressions of the SMRs on calendar year are presented in Table IV. Roughly forty percent of them were appropriate for tabulation according to the criteria above. These in general indicated trends similar to those seen in the before/after comparisons. There were statistically significant increasing trends for all malignancies combined around Trawsfynydd, Springfields, Capenhurst, and Harwell. Of these, the slope for Trawsfynydd was the largest, a 2.5% per year rise in relative mortality. Springfield also had a significantly rising slope for leukaemia, 1.5% per year. Both Trawsfynydd and Sizewell had larger estimated slopes for leukaemia, but these were not statistically different from zero. For both the conventional and nuclear electricity groups, the pooled estimates of the slopes were all close to zero, indicating virtually no linear trend in relative mortality.

### Discussion

This report has considered temporal trends in relative cancer mortality around 14 nuclear and 5 non-nuclear facilities in England and Wales. No attempt has been made to draw conclusions from

**Table II** Expected deaths and $O/E$ ratios[a] for baseline and later periods, by facility and cause of death.

| | **All causes** | | | | **All malignancies** | | | | **Cancer of the stomach** | | | |
|---|---|---|---|---|---|---|---|---|---|---|---|---|
| | *Baseline* | | *15+years after start-up* | | *Baseline* | | *15+years after start-up* | | *baseline* | | *15+years after start-up* | |
| *Conventional electricity generating facilities* | *E* | *O/E* | *E* | *O/E* | *E* | *O/E* | *E* | *O/E* | *E* | *O/E* | *E* | *O/E* |
| Little Barford | 330.56 | 0.87 | 2721.15 | 0.86 | 49.95 | 1.24 | 537.60 | 0.93[b] | 9.13 | 0.77 | 58.68 | 0.70 |
| Fleetwood | 2859.17 | 1.15 | 9299.88 | 1.04[c] | 492.88 | 1.08 | 1956.38 | 1.01 | 81.53 | 1.15 | 195.33 | 1.06 |
| Portishead | 319.64 | 0.99 | 1048.27 | 1.01 | 52.05 | 1.15 | 215.19 | 1.03 | 8.70 | 1.15 | 20.84 | 0.82 |
| Richborough | 8622.70 | 0.86 | 6505.15 | 0.98[c] | 1505.42 | 0.93 | 1366.92 | 0.98 | 225.75 | 0.83 | 126.63 | 1.03 |
| South Denes | 3368.92 | 0.97 | 5676.81 | 1.06[c] | 577.63 | 1.17 | 1172.94 | 1.06[b] | 92.55 | 0.99 | 113.98 | 1.35[b] |
| | *Baseline* | | *10+years after start-up* | | *Baseline* | | *10+years after start-up* | | *Baseline* | | *10+years after start-up* | |
| *Nuclear electricity generating facilities* | *E* | *O/E* | *E* | *O/E* | *E* | *O/E* | *E* | *O/E* | *E* | *O/E* | *E* | *O/E* |
| Bradwell | 2150.06 | 0.97 | 4419.27 | 0.89[c] | 374.39 | 0.99 | 904.99 | 0.88 | 55.71 | 0.97 | 87.68 | 0.83´ |
| Berkeley | 3137.61 | 0.98 | 6770.80 | 0.90[c] | 575.56 | 0.90 | 1453.18 | 0.87 | 82.58 | 0.84 | 136.44 | 0.88 |
| Hinkley | 3895.36 | 0.97 | 4529.83 | 0.99 | 702.85 | 0.96 | 966.52 | 1.00 | 95.54 | 0.91 | 89.30 | 1.12 |
| Dungeness | 227.48 | 0.70 | 257.12 | 1.12[c] | 41.31 | 0.90 | 56.86 | 1.25 | 5.65 | 0.53 | 5.22 | 1.15 |
| Trawsfynydd | 466.56 | 1.31 | 440.03 | 1.11[b] | 83.67 | 1.09 | 95.51 | 1.13 | 11.44 | 1.57 | 8.78 | 1.14 |
| Sizewell | 635.93 | 0.87 | 483.16 | 1.00[b] | 111.05 | 0.94 | 99.89 | 0.95 | 15.08 | 1.00 | 9.29 | 1.29 |
| | *Baseline* | | *15+years after start-up* | | *Baseline* | | *15+years after start-up* | | *Baseline* | | *15+years after start-up* | |
| *Other nuclear facilities* | *E* | *O/E* | *E* | *O/E* | *E* | *O/E* | *E* | *O/E* | *E* | *O/E* | *E* | *O/E* |
| Amersham | 3915.52 | 0.86 | 26858.20 | 0.83 | 608.08 | 0.95 | 5272.77 | 0.93 | 110.05 | 0.90 | 607.85 | 0.69[b] |
| Aldermaston | 1294.07 | 0.82 | 4937.28 | 0.82 | 202.58 | 0.89 | 1030.34 | 0.88 | 36.06 | 0.97 | 1.01.40 | 0.65 |
| Springfields | 7786.83 | 1.20 | 29321.81 | 1.16[c] | 1230.22 | 1.09 | 5975.77 | 1.04 | 220.53 | 1.16 | 639.03 | 1.23 |
| Capenhurst | 1348.42 | 1.01 | 5301.41 | 1.04 | 227.27 | 1.03 | 1152.31 | 1.17 | 38.00 | 0.87 | 108.40 | 1.25 |
| Harwell | 2455.24 | 0.88 | 12613.49 | 0.94[b] | 372.99 | 0.82 | 2520.82 | 0.95[b] | 67.79 | 0.74 | 268.43 | 0.83 |
| Winfrith | 1310.02 | 0.95 | 1809.97 | 0.86[b] | 233.90 | 1.02 | 395.64 | 0.89 | 34.98 | 1.09 | 36.23 | 0.83 |
| Windscale | 2133.69 | 1.18 | 7252.89 | 1.07[c] | 330.29 | 1.03 | 1503.05 | 0.95 | 60.03 | 1.28 | 154.65 | 1.15 |

| | **Cancer of the lung** | | | | **Cancer of the female breast** | | | |
|---|---|---|---|---|---|---|---|---|
| | *Baseline* | | *15+years after start-up* | | *Baseline* | | *15+years after start-up* | |
| *Conventional electricity generating facilities* | *E* | *O/E* | *E* | *O/E* | *E* | *O/E* | *E* | *O/E* |
| Little Barford | — | — | 137.25 | 0.95 | 4.91 | 1.43 | 49.32 | 0.91 |
| Fleetwood | 80.98 | 0.96 | 531.46 | 1.01 | 45.44 | 1.21 | 178.66 | 1.07 |
| Portishead | 7.93 | 0.50 | 56.24 | 0.80 | 4.83 | 1.03 | 20.96 | 0.95 |
| Richborough | 302.54 | 0.99 | 365.02 | 0.93 | 138.31 | 0.92 | 128.43 | 1.04 |
| South Denes | 100.10 | 1.25 | 314.33 | 1.21 | 53.62 | 1.19 | 109.49 | 1.10 |
| | *Baseline* | | *15+years after start-up* | | *Baseline* | | *15+years after start-up* | |
| *Nuclear electricity generating facilities* | *E* | *O/E* | *E* | *O/E* | *E* | *O/E* | *E* | *O/E* |
| Bradwell | 79.28 | 0.92 | 244.64 | 0.84 | 31.36 | 1.05 | 81.45 | 1.02 |
| Berkeley | 128.69 | 0.75 | 399.96 | 0.78 | 50.02 | 1.30 | 134.90 | 0.88[b] |
| Hinkley | 160.81 | 0.82 | 260.41 | 0.83 | 64.39 | 1.17 | 91.73 | 1.09 |
| Dungeness | 10.15 | 0.99 | 16.26 | 1.23 | 3.27 | 0.92 | 4.87 | 1.64 |
| Trawsfynydd | 19.19 | 1.04 | 25.66 | 1.05 | 7.52 | 1.33 | 9.29 | 1.29 |
| Sizewell | 25.09 | 0.76 | 26.53 | 0.83 | 1.0.02 | 1.60 | 9.38 | 1.39 |

**Table II (continued)**

| Other nuclear facilities | Cancer of the lung | | | | Cancer of the female breast | | | |
|---|---|---|---|---|---|---|---|---|
| | Baseline | | 15+years after start-up | | Baseline | | 15+years after start-up | |
| | E | O/E | E | O/E | E | O/E | E | O/E |
| Amersham | — | — | 1290.66 | 0.89 | 61.78 | 1.23 | 507.73 | 1.11 |
| Aldermaston | 35.67 | 0.62 | 276.56 | 0.82 | 18.54 | 1.24 | 96.33 | 0.81 |
| Springfields | 240.89 | 0.92 | 1546.11 | 1.07[b] | 127.45 | 1.08 | 572.30 | 0.89 |
| Capenhurst | 44.88 | 1.03 | 312.57 | 1.22 | 20.65 | 1.07 | 111.86 | 1.08 |
| Harwell | 74.39 | 0.69 | 652.89 | 0.88 | 36.24 | 0.94 | 234.45 | 1.03 |
| Winfrith | 48.75 | 0.62 | 110.27 | 0.77 | 19.93 | 1.00 | 35.80 | 1.20 |
| Windscale | 66.37 | 0.71 | 399.08 | 0.80 | 30.92 | 1.04 | 140.19 | 0.85 |

| Conventional electricity generating facilities | Cancer of the uterus | | | | Leukaemia | | | |
|---|---|---|---|---|---|---|---|---|
| | Baseline | | 15+years after start-up | | Baseline | | 15+years after start-up | |
| | E | O/E | E | O/E | E | O/E | E | O/E |
| Little Barford | 2.70 | 0 | 17.72 | 1.24 | — | — | 15.35 | 0.78 |
| Fleetwood | 22.44 | 1.38 | 58.22 | 0.86[b] | 10.92 | 1.10 | 47.36 | 1.01 |
| Portishead | 2.36 | 0.85 | 6.73 | 0.89 | 1.14 | 0.88 | 5.81 | 1.38 |
| Richborough | 61.93 | 0.74 | 39.56 | 0.81 | 34.85 | 1.00 | 32.70 | 1.07 |
| South Denes | 24.99 | 1.52 | 34.68 | 1.18 | 13.34 | 0.68 | 29.04 | 0.72 |
| **Nuclear electricity generating facilities** | Baseline | | 10+years after start-up | | Baseline | | 10+years after start-up | |
| | E | O/E | E | O/E | E | O/E | E | O/E |
| Bradwell | 14.06 | 0.64 | 25.80 | 0.54 | 9.12 | 0.88 | 23.38 | 0.68 |
| Berkeley | 22.55 | 0.89 | 42.56 | 0.96 | 15.48 | 1.36 | 39.08 | 0.90 |
| Hinkley | 27.36 | 1.06 | 28.27 | 1.03 | 18.75 | 0.43 | 24.75 | 0.69 |
| Dungeness | 1.39 | 0 | 1.51 | 0.66 | 1.08 | 0 | 1.49 | 0.67 |
| Trawsfynydd | 3.19 | 0.31 | 2.87 | 2.09[b] | 2.20 | 0 | 2.39 | 2.09[b] |
| Sizewell | 4.14 | 1.45 | 2.89 | 0.35 | 2.77 | 0.36 | 2.47 | 2.02 |
| **Other nuclear facilities** | Baseline | | 15+years after start-up | | Baseline | | 15+years after start-up | |
| | E | O/E | E | O/E | E | O/E | E | O/E |
| Amersham | 34.25 | 0.91 | 190.07 | 0.74 | — | — | 141.00 | 1.00 |
| Aldermaston | 9.81 | 0.51 | 32.09 | 0.90 | 4.88 | 1.23 | 29.05 | 0.93 |
| Springfields | 71.80 | 1.16 | 202.14 | 1.21 | 35.29 | 0.65 | 156.83 | 0.95 |
| Capenhurst | 10.84 | 1.20 | 36.90 | 1.41 | 6.84 | 1.17 | 34.75 | 1.15 |
| Harwell | 20.09 | 0.90 | 82.52 | 0.53 | 10.74 | 0.56 | 70.41 | 0.94 |
| Winfrith | 9.17 | 0.87 | 11.05 | 1.27 | 6.08 | 1.65 | 10.22 | 0.69 |
| Windscale | 17.40 | 1.15 | 48.24 | 0.95 | 9.54 | 1.15 | 40.58 | 0.96 |

[a]SMRs = $O/E$ ratios × 100.
[b]Significantly different from baseline, $P < 0.05$.
[c]Significantly different from baseline, $P < 0.01$.
—Baseline data unavailable.

**Table III** Expected childhood[a] deaths and *O/E* ratios[b] for early and later periods, by facility and cause of death.

| | *All causes* | | | | *All malignancies* | | | | *Leukaemia* | | | |
|---|---|---|---|---|---|---|---|---|---|---|---|---|
| | *1963–1970* | | *1972–1979* | | *1963–1970* | | *1972–1979* | | *1963–1970* | | *1972–1979* | |
| | *E* | *O/E* | *E* | *O/E* | *E* | *O/E* | *E* | *O/E* | *E* | *O/E* | *E* | *O/E* |
| Bradwell | 118.03 | 0.98 | 120.97 | 0.78 | 4.55 | 1.98 | 4.89 | 1.02 | 1.95 | 2.57 | 2.03 | 1.48 |
| Berkeley | 261.90 | 1.00 | 241.37 | 0.70[c] | 10.29 | 1.46 | 10.37 | 0.87 | 4.41 | 2.04 | 4.33 | 1.16 |
| Hinkley | 218.21 | 0.83 | 180.20 | 0.75 | 8.90 | 1.01 | 8.02 | 1.12 | 3.82 | 0.78 | 3.35 | 1.79 |
| Dungeness | 16.12 | 0.74 | 14.54 | 0.41 | 0.63 | 0.00 | 0.61 | 1.65 | 0.27 | 0.00 | 0.25 | 0.00 |
| Trawsfynydd | 20.86 | 0.72 | 14.65 | 1.02 | 0.84 | 0.00 | 0.67 | 2.98 | 0.36 | 0.00 | 0.28 | 3.56 |
| Sizewell | 25.44 | 0.71 | 18.23 | 0.88 | 1.00 | 1.99 | 0.78 | 0.00 | 0.43 | 2.32 | 0.33 | 0.00 |

[a]0–14 years of age.
[b]SMRs = *O/E* ratios × 100.
[c]Statistically different from SMR for early period, $P<0.001$.

**Table IV** Regression coefficients of SMRs on calendar year by site and cause of death, from start-up onwards.

| | *All causes* | *All malig.* | *Stomach* | *Lung* | *Female breast* | *Uterus* | *Leukaemia* |
|---|---|---|---|---|---|---|---|
| *Conventional electricity generating facilities* | 0.06[a] | 0.03 | 0.71 | 0.22[a] | 0.17 | 0.35 | 0.21 |
| Little Barford | — | — | — | 2.16[b] | — | 4.15[b] | — |
| Fleetwood | −0.31 | 0.30 | — | 1.12[b] | — | — | — |
| Portishead | — | 0.66 | — | — | — | 4.23 | — |
| Richborough | 0.33 | — | 0.99 | −1.07[c] | — | — | — |
| South Denes | 0.59[c] | — | 2.55[b] | — | — | −1.66 | — |
| *Nuclear electricity generating facilities* | 0.10[a] | 0.01 | 0.40 | 0.32 | −0.59 | −2.31 | −0.19[a] |
| Bradwell | — | −0.89 | — | — | −2.59 | −6.84[b] | −5.70[b] |
| Berkeley | — | — | — | — | — | — | — |
| Hinkley | 0.26 | — | — | — | — | — | — |
| Dungeness | 3.89[c] | 2.57 | 6.77 | — | — | — | — |
| Trawsfynydd | — | 2.49[b] | −3.71 | 4.00 | — | 6.93 | 9.64 |
| Sizewell | 1.66[b] | 1.66 | 4.53 | 1.87 | 10.66 | — | 14.98 |
| *Other nuclear facilities* | | | | | | | |
| Amersham | 0.09 | — | −0.51 | — | — | — | — |
| Aldermaston | — | — | −0.99 | — | — | — | — |
| Springfields | 0.10 | 0.27[b] | 0.76[b] | 0.56[b] | — | — | 1.50[b] |
| Capenhurst | 0.31 | 0.79[b] | — | 1.26[b] | — | — | 2.18 |
| Harwell | 0.32[c] | 0.71[c] | — | 1.18[c] | — | −1.19 | — |
| Winfrith | −0.15 | — | 1.25 | — | — | 4.43 | −3.02 |
| Windscale | — | — | — | — | — | — | −2.04 |

[a]Statistically significant heterogeneity of slopes, $P<0.05$.
[b]Significantly different from 0, $P<0.05$.
[c]Significantly different from 0, $P<0.01$.
—$r^2<0.04$.

**Table V** Regression coefficients of SMRs on calendar year, aggregate of rural districts, 1950–1973.

| *All causes* | *All malig.* | *Stomach* | *Lung* | *Female breast* | *Uterus* | *Leukaemia* |
|---|---|---|---|---|---|---|
| 0.14 | 0.13 | −0.24 | 0.65 | 0.15 | 0.31 | 0.18 |

generally high or generally low SMRs in any locale because of the known geographic variations in mortality (Gardner *et al.*, 1983).

Though virtually any malignancy can be caused by radiation (Advisory Committee on the Biological Effects of Ionizing Radiation, 1980), data presented here regarding several of the individual malignancies are especially difficult to interpret. The absence of cigarette smoking data makes hazardous any analysis of lung cancer trends. Similarly, the pronounced decline in stomach cancer mortality during the study period implies important environmental changes that could relate to locale. The designation "uterine cancer" obscures differences between the endometrial and cervical sites included. For these reasons, of the malignancies considered here it is most sensible to focus on leukaemia and breast cancer. All malignancies combined is also of potential interest, though over the study period it became increasingly dominated numerically by lung cancer.

The use of England and Wales death rates for standardization did not cause any obvious difficulties in our largely rural population. The SMRs for the aggregate of all the rural districts in England and Wales showed virtually no trend over time (Table V). This indicates that there are not to be expected important increases in local cancer SMRs simply from national changes in the nature of rural populations or rural diagnostic fashion. Of course such changes could well occur in a single small area and thus lead to artifactual findings around a particular establishment.

The pooled regression analyses regarding the nuclear and conventional electric facilities indicate little cause for concern: there were no increasing trends greater than 0.71% per year in relative mortality. This overall stability in the cancer SMRs around both types of electric plants provides some evidence that, in general, the areas around these facilities have had mortality changes that do not differ substantially from the national experience.

Overall, the data for the individual facilities also indicate no general pattern of rising SMRs. There are some increasing and some decreasing trends, as might be expected from a large data set with no underlying effect. Most of these trends are of small magnitude. Some of the specific data deserve comment, however. Around Dungeness, Sizewell, and Harwell there were increasing SMRs for most of the causes of death considered. This suggests either a general worsening of relative mortality or an artifact in the data. In contrast, around Trawsfynydd there were increases in relative mortality from all malignancies (and leukaemia and cancer of the uterus) in the face of decreasing all cause relative mortality. This pattern is less likely to be due to artifacts in the data, and was also seen in the area around Springfields. Both of these nuclear facilities discharge liquid effluents into fresh water, and have radioactive discharges somewhat higher than many other plants, though still well within accepted limits (Department of the Environment, 1980).

Conspicuously free of increasing SMRs is the area around Windscale, despite that facility's large radiation releases into the atmosphere and into the Irish Sea (Taylor & Webb, 1978; Department of the Environment, 1980). These negative findings contrast with those of some other recent analyses (Yorkshire Television, November 1st, 1983; Gardner & Winter, 1984*a*; Urquhart *et al.*, 1984) which considered childhood malignancies in smaller areas around the plant and therefore differed from the analysis presented here. Also free of important trends are the environs of Berkeley and Oldbury, the only small area considered here that has two sites within it.

Among the five conventional fuel stations included in the analysis, Fleetwood and Little Barford use coal for at least one generator. Use of this fuel has been associated with small radiation releases (McBride *et al.*, 1978; Bauman & Howat, 1981) but there was no indication in this study that these two stations had an associated pattern of increasing cancer SMRs.

Based on routinely-collected mortality data, this report is subject to many limitations. The inaccuracies possible in death certificate information are well known, though this is apparently less of a problem for neoplasms than for other diseases (MacMahon & Pugh, 1970; Doll & Peto, 1981). However, awareness of the association between radiation and cancer has been growing since World War II, and it is possible that physicians near certain nuclear facilities might have become increasingly biased towards certifying malignancies as causes of death. An example of other artifacts possible with death certification data has recently been published (Gardner & Winter, 1984*a, b*).

Since the actual populations involved in the SMR calculations differ from year to year (due to births, deaths, and migration), it is difficult to exclude an artifactual basis for any apparent trends in mortality. For example, the construction of a major facility may encourage certain types of people to emigrate away from the surrounding area, and others to move in. The resulting changes in the population could be associated with mortality trends in the absence of any other direct effect of the plant.

Inaccuracies in the local population estimates are another potential source of error. In 1946, for example, the rapid demobilization caused particular difficulties in using mid-year population estimates

(Registrar General, 1951). Furthermore, the age–sex proportions used here were based on interpolation between censuses, and could be subject to error. The aging of the population of England and Wales in the years after 1971 (Office of Population Censuses and Surveys, 1974 *et seq.*) implies that the use of 1971 census data for the age/sex proportions in the following years may have resulted in slight underestimates of the size of the local populations in the older age groups. Given the high cancer mortality in the older populations, such a systematic error could induce artifactually rising SMRs over the study period. Finally, even the total population estimates after 1973 involve some uncertainty; a systematic under-estimate in these later years could also result in artifactually rising SMRs.

The large number of statistics calculated and examined in this study opens the possibility that some trends may have been identified by chance alone in the absence of any changes in the underlying force of mortality. Reliance on patterns of trends in different SMRs makes this less likely, but cannot completely protect against it. Conversely, the lack of statistical power associated with small numbers of observed deaths opens the possibility that some real changes in mortality might be missed. This is of particular concern for childhood mortality and the less common causes of death over all ages (e.g. leukaemia).

Small numbers of deaths may also call into question the use of SMRs in regression analysis, which assumes continuous variables (Armitage, 1971). This may hamper interpretation of the less common causes of death such as leukaemia. As noted above, this group of malignancies was of particular interest around Trawsfynydd and Springfields because of the contrast with all cause mortality. To confirm the conclusions previously reached about the mortality patterns around these facilities, the leukaemia trends around them were re-examined using a generalized linear model (Nelder & Wedderburn, 1974) and the Poisson distribution. This assumes that for a particular facility the number of observed deaths in a given year from a particular cause is distributed as a Poisson variable (Armitage, 1971) with an expected value equal to $Ee^{a+bx}$ (Here $E$ is the expected number of deaths, $x$ is the calendar year, and $a$ and $b$ are parameters to be estimated.) These models confirm a statistically significant increasing trend in relative leukaemia mortality around those two sites.

In summary, the data described here indicate no generalized trend of rising cancer mortality in small areas around the major nuclear facilities in England and Wales. These results are similar to those found in analogous studies from other areas (Tokuhata & Smith, 1981; Patrick, 1977; Geary *et al.*, 1979; Enstrom, 1983), and the lack of an overall effect is consistent with the generally small radiation releases reported (Department of the Environment, 1980). Despite the limitations to the data, it seems likely that over all, the facilities investigated here have not had an important impact on the cancer mortality of surrounding populations. It is possible, however, that a longer period of observation might allow detection of some effects as exposure and follow-up continued. In view of all these uncertainties, monitoring should continue, and the few patterns suggestive of a radiation effect around individual facilities should be investigated in more detail.

(Much of this work was done while the author was a Milbank Scholar in the Department of Community Medicine and General Practice, Oxford University, Oxford, England, during 1981–1982).

The author would like to acknowledge the support of numerous colleagues. Peter Smith was particularly helpful throughout the investigation. Thanks are also due to Sir Richard Doll and Paula Cook-Mozaffari for their encouragement and advice; to the Medical Statistics Division at OPCS for help with their data; and to Richard Stratton, Alice Schori, Jane Barrett, Peter Smith, David Dunn and Karen Nielson for help with statistics and computations.

This work was partially supported by the Milbank Memorial Fund, and by BRSG2 S07 RR05392-22 awarded by the Biomedical Research Support Grant Program, Division of Research Resources, National Institutes of Health.

### References

ADVISORY COMMITTEE ON THE BIOLOGICAL EFFECTS OF IONIZING RADIATION (1980). *The Effects on Populations of Exposure to Low Levels of Ionizing Radiation*. Washington DC: National Academy of Sciences.

ARMITAGE, P. (1971). *Statistical Methods in Medical Research*. Oxford: Blackwell.

BAILAR, J. & EDERER, F. (1964). Significance factors for the ratio of a Poisson variable to its expectation. *Biometrics*, **20**, 639.

BAUMAN, A. & HOWAT, D. (1981). The impact of natural radioactivity from a coal fired power plant. *Sci. Total Environ.*, **17**, 75.

COMMISION OF THE EUROPEAN COMMUNITIES (1979). Methodology for evaluating the radiological consequences of radioactive effluents released in normal operations.

COMPTROLLER GENERAL (1981). *Problems in Assessing the Cancer Risks of Low-Level Radiation Exposure*. Washington: G.A.O.

DAVIES, J.M. & CHILVERS, C. (1980). The study of mortality variations in small administrative areas of England and Wales, with reference to cancer. *J. Epidemiol Comm. Health*, **34**, 87.

DEPARTMENT OF THE ENVIRONMENT (1980). *Annual Survey of Radioactive Discharges in Great Britain, 1979*. London: Department of the Environment.

DOLL, R. & PETO, R. (1981). The causes of cancer. *J. Natl. Cancer Inst.*, **66**, 1191.

ENSTROM, J.E. (1983). Cancer mortality patterns around the San Onofre power plant, 1960–1978. *Am. J. Pub. Health*, **73**, 83.

GARDNER, M.J., WINTER, P.D., TAYLOR, C.P. ACHESON, E.D. (1983). *Atlas of Cancer Mortality for England and Wales 1968–1978*. Chichester: Wiley.

GARDNER, M.J. & WINTER, P. D. (1984*a*). Mapping small area cancer mortality: a residential coding story. *J. Epidemiol Comm. Health*, **38**, 81.

GARDNER, M.J. & WINTER, P.D. (1984*b*). Mortality in Cumberland during 1958–78 with reference to cancer in young people around Windscale. *Lancet*, **i**, 216 (letter).

GEARY, C.G., BENN, R.T. & LECK, I. (1979). Incidence of myeloid leukaemia in Lancashire. *Lancet*, **ii**, 549.

GOWING, E. (1974). *Independence and Deterence: Britain and Atomic Energy, 1945–1952*. New York: MacMillan.

LAND, C.E. (1980). Estimating cancer risks from low doses of ionizing radiation. *Science*, **209**, 1197.

LEVINE, R.J., SYMONS, M.J., BALOGH, S.A. & others. (1980). A method for monitoring the fertility of workers. *J. Occ. Med.*, **22**, 781.

MACMAHON, B. & PUGH, T.F. (1970). *Epidemiology*. Boston: Little Brown.

McBRIDE, J.P., MOORE, R.E., WITHERSPOON, J.P. & BLANCO, R.E. (1978). Radiological impact of airborne effluents of coal and nuclear plants. *Science*, **202**, 1045.

NELDER, J.A. & WEDDERBURN, R.W.M. (1974). Generalized linear models. *JRSS*(A), **135**, 370.

OFFICE OF POPULATION CENSUSES AND SURVEYS (1974 and se.). *Population Estimates, Series PP1*. London: HMSO.

OFFICE OF POPULATION CENSUSES AND SURVEYS (1982). *Preliminary Report on Towns*. London: HMSO.

OFFICE OF POPULATION CENSUSES AND SURVEYS (1975). *Reorganization of Local Government Areas. Correlation of New and Old Areas*. London: HMSO.

PATRICK, C.H. (1977). Trends in public health in the population near nuclear facilities: a critical assessment. *Nuclear Safety*, **18**, 647.

POPULATION STATISTICS DIVISION, OFFICE OF POPULATION CENSUSES AND SURVEYS (1979). *Geographical Area Units. Population Trends 15*. London: HMSO, p. 16.

REGISTRAR GENERAL (1951). *Statistical Review of England and Wales for the Two Years 1946–47*. Text, Vol. 1, Medical. London: HMSO, p. 7.

REGISTRAR GENERAL (1934 and seq.). *Statistical Review of England and Wales*. London: HMSO.

TAYLOR, F.E. & WEBB, G.A.M. (1978). *Radiation Exposure of the UK Population*. Didcot: National Radiological Protection Board.

TOKUHATA, K.G. & SMITH, M.W. (1981). History of health studies around nuclear facilities: a methodological consideration. *Environmental Res.*, **25**, 75.

UNITED KINGDOM, ATOMIC ENERGY AUTHORITY (1979). *Atomic Energy in Britain, 1939–1975*. Harwell: AERE.

URQUHART, J., PALMER, M. & CUTLER, J. (1984). Cancer in Cumbria: the Windscale connection. *Lancet*, **i**, 217 (letter).

WORLD LIST OF NUCLEAR POWER PLANTS (1983). *Nuclear News*, **26**, 71.


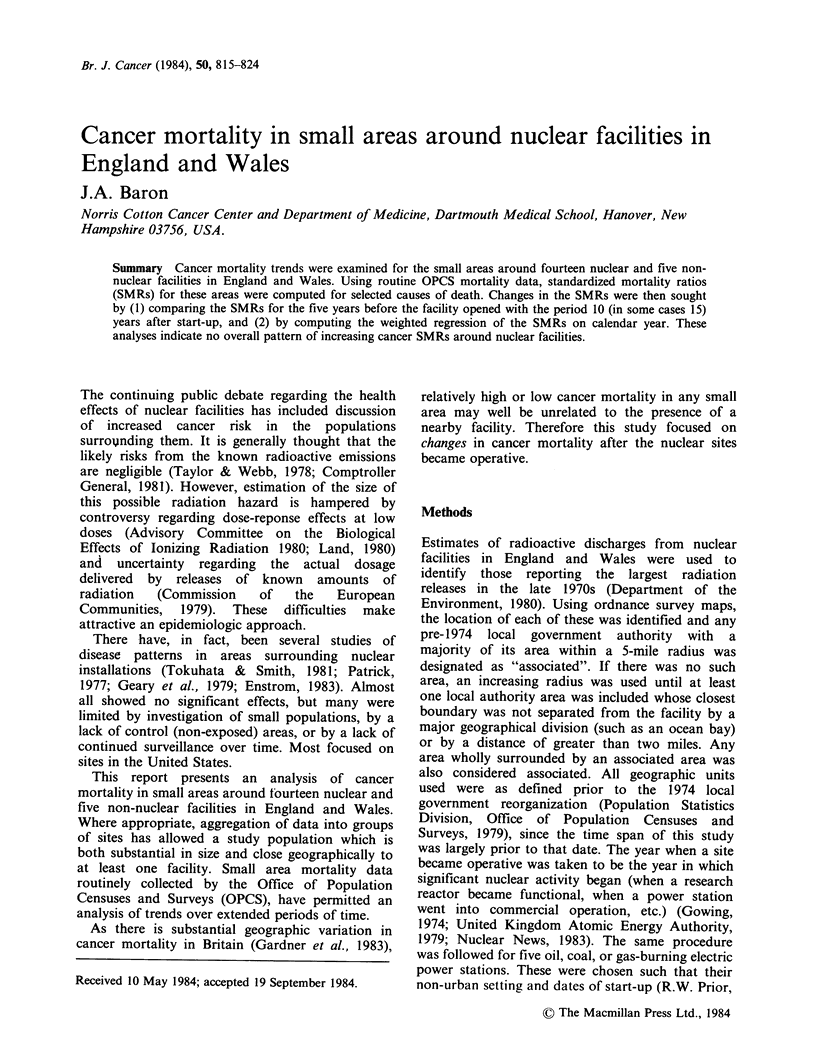

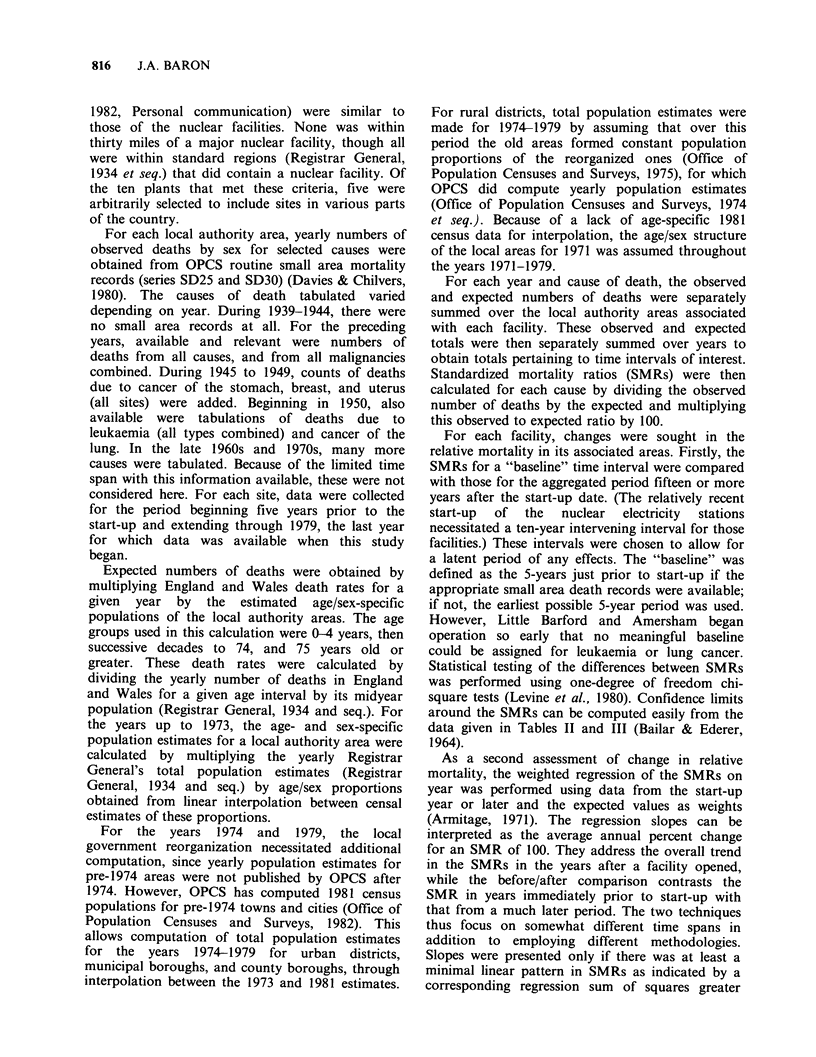

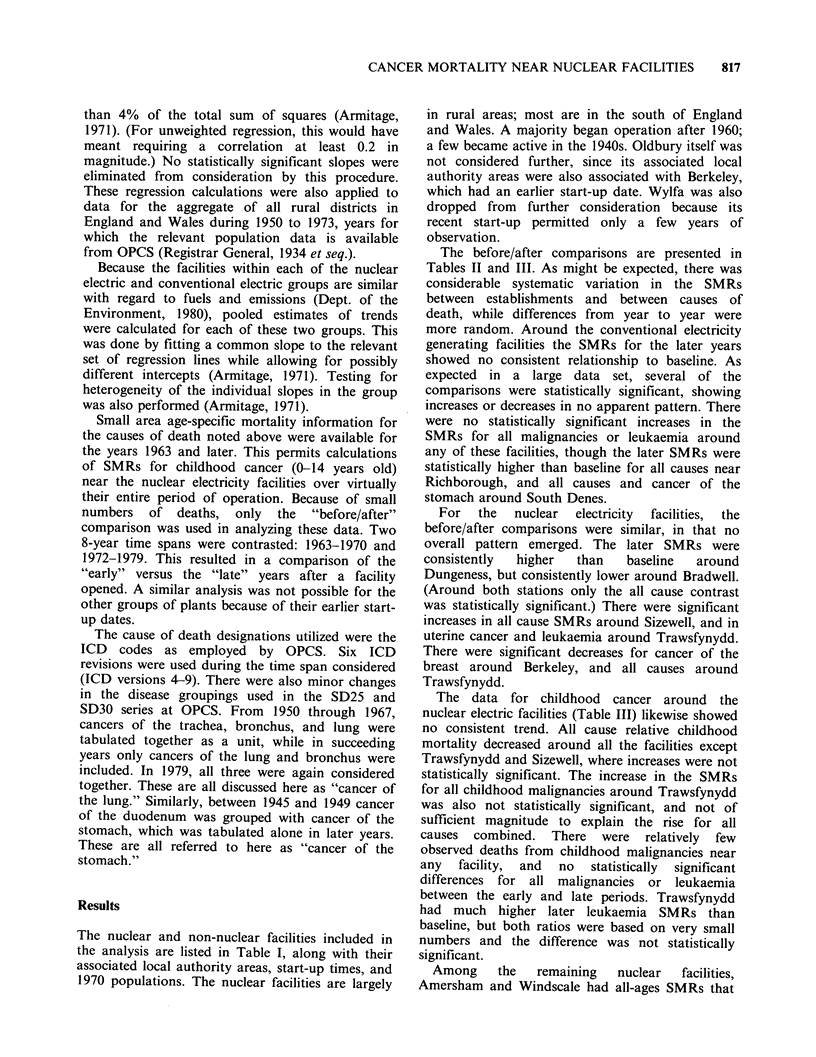

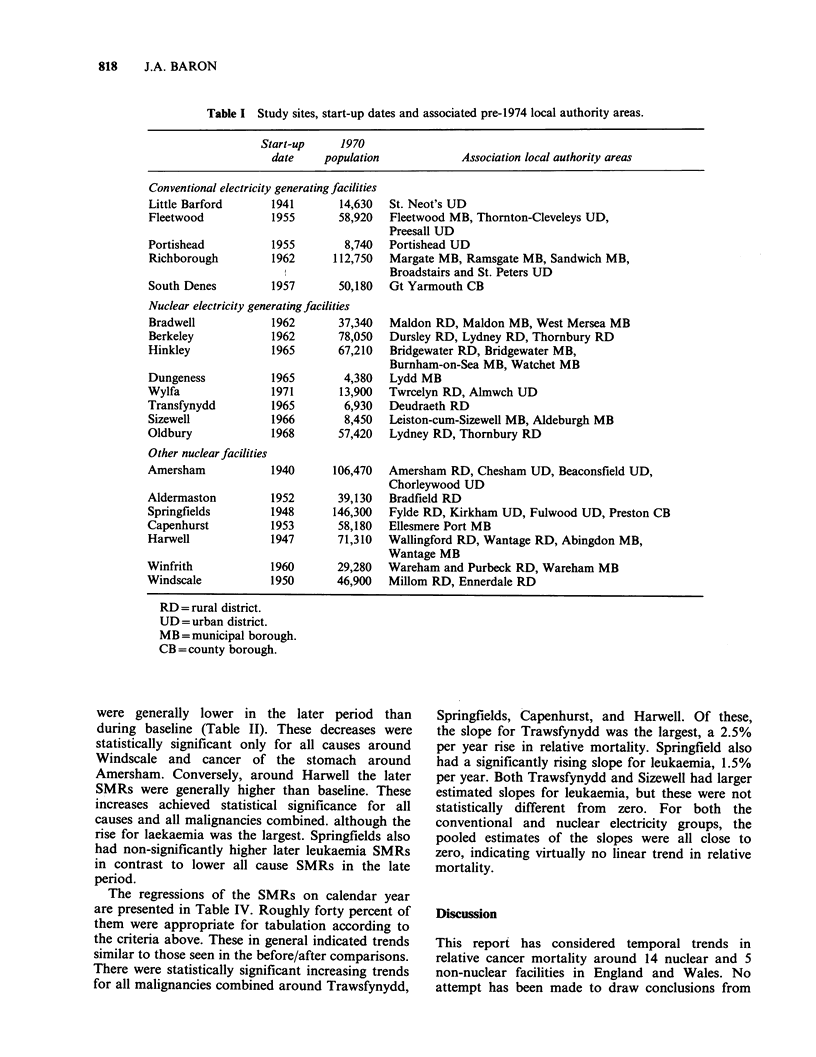

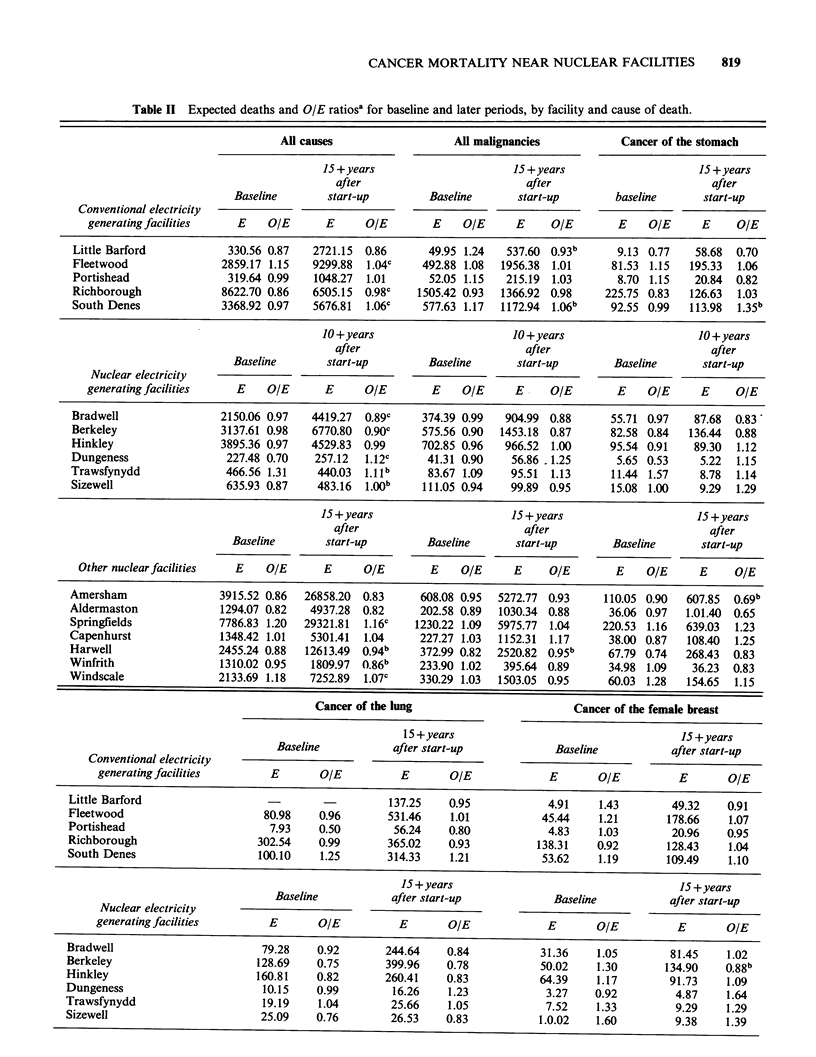

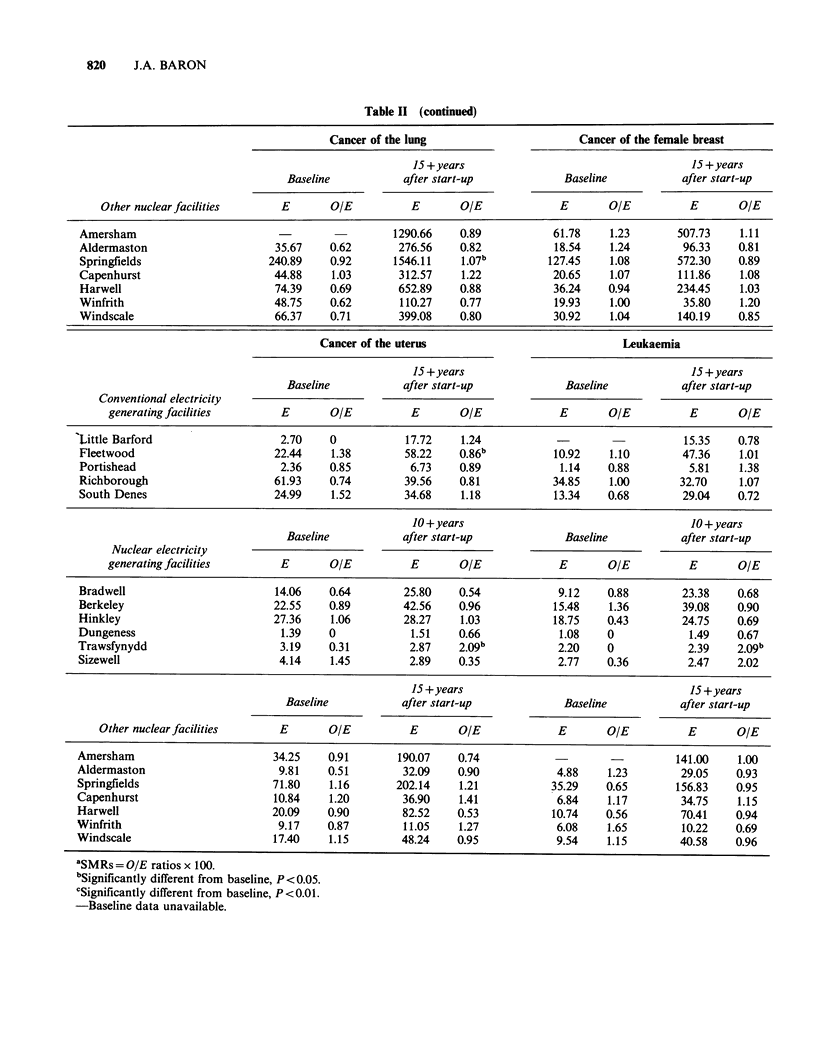

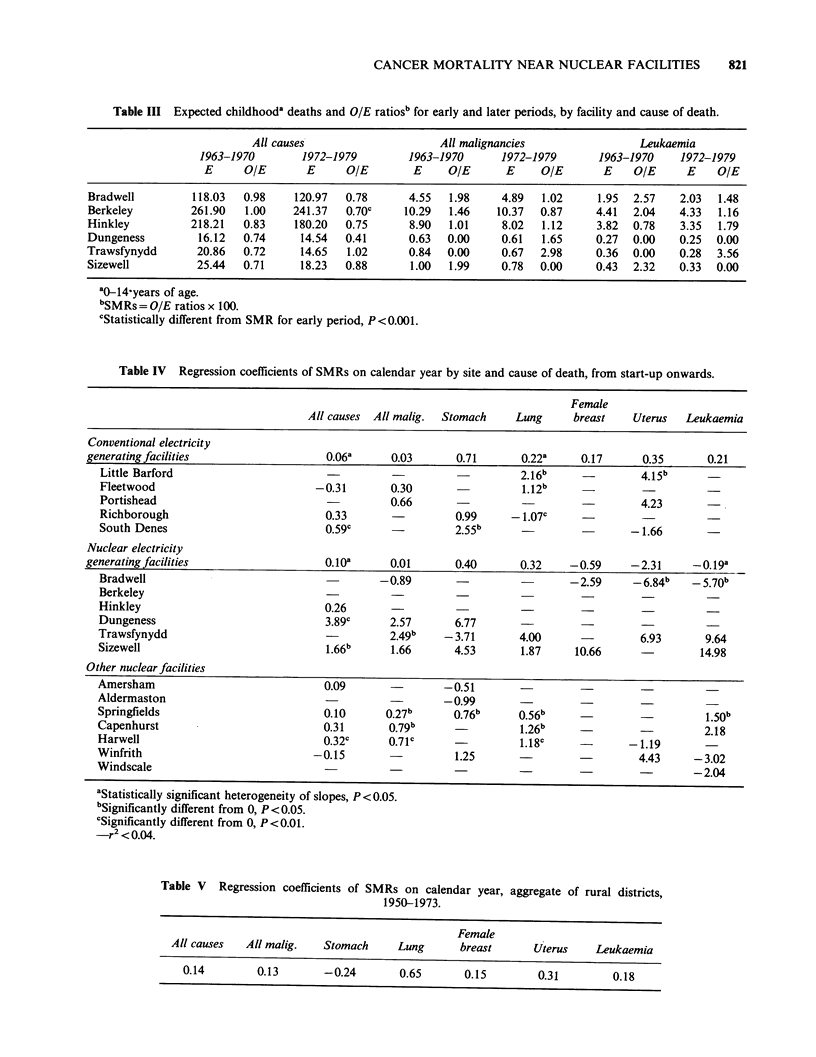

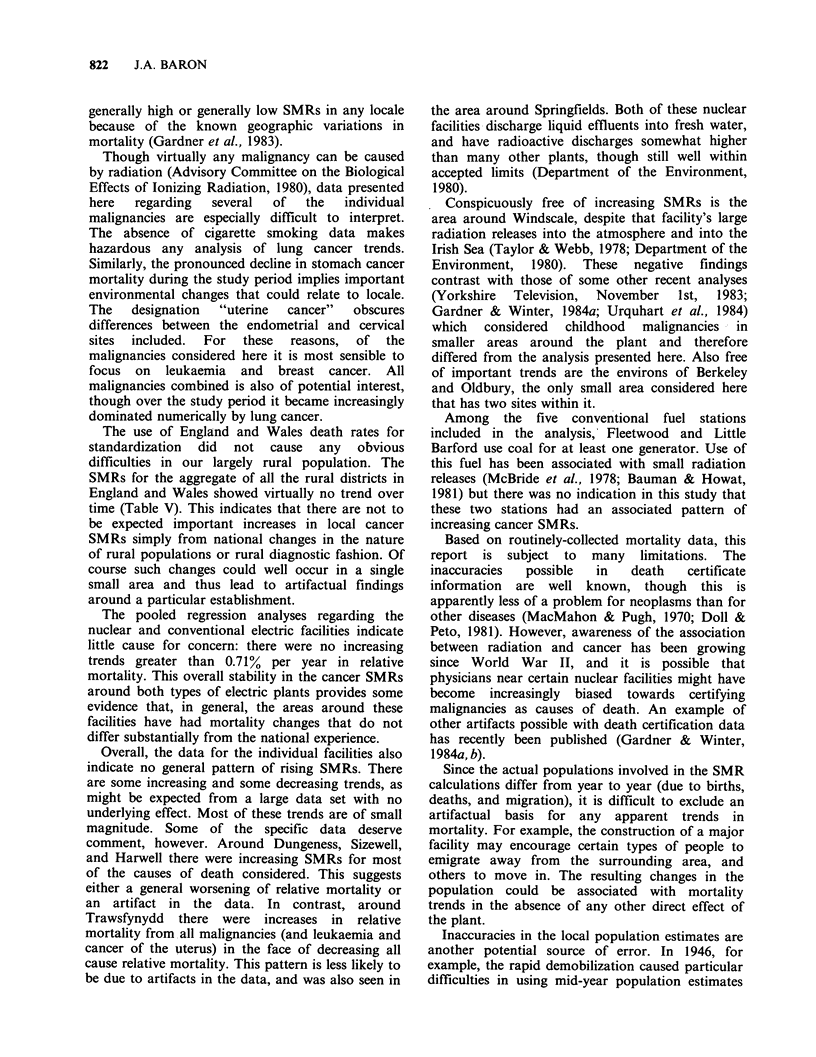

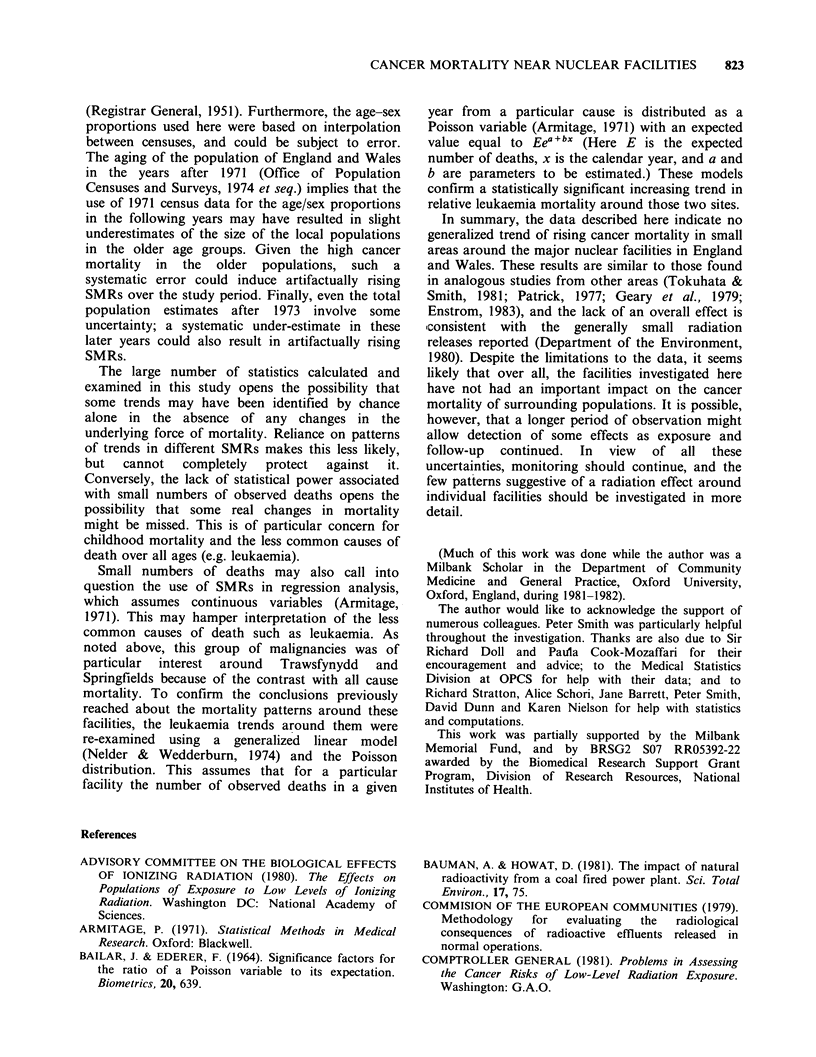

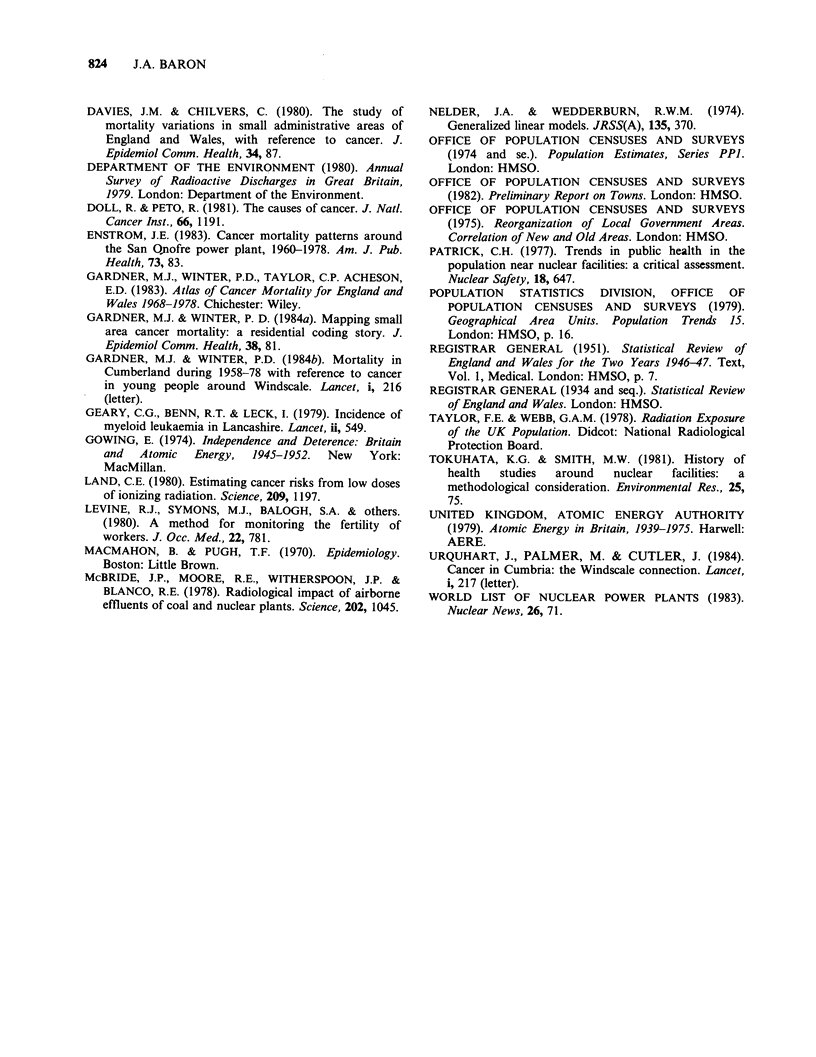


## References

[OCR_00926] Bauman A., Horvat D. (1981). The impact of natural radioactivity from a coal-fired power plant.. Sci Total Environ.

[OCR_00944] Davies J. M., Chilvers C. (1980). The study of mortality variations in small administrative areas of England and Wales, with special reference to cancer.. J Epidemiol Community Health.

[OCR_00955] Doll R., Peto R. (1981). The causes of cancer: quantitative estimates of avoidable risks of cancer in the United States today.. J Natl Cancer Inst.

[OCR_00959] Enstrom J. E. (1983). Cancer mortality patterns around the San Onofre nuclear power plant, 1960-1978.. Am J Public Health.

[OCR_00969] Gardner M. J., Winter P. D. (1984). Mapping small area cancer mortality: a residential coding story.. J Epidemiol Community Health.

[OCR_00980] Geary C. G., Benn R. T., Leck I. (1979). Incidence of myeloid leukaemia in Lancashire.. Lancet.

[OCR_00989] Land C. E. (1980). Estimating cancer risks from low doses of ionizing radiation.. Science.

[OCR_00993] Levine R. J., Symons M. J., Balogh S. A., Arndt D. M., Kaswandik N. T., Gentile J. W. (1980). A method for monitoring the fertility of workers. 1. Method and pilot studies.. J Occup Med.

[OCR_01002] McBride J. P., Moore R. E., Witherspoon J. P., Blanco R. E. (1978). Radiological impact of airborne effluents of coal and nuclear plants.. Science.

[OCR_01049] Tokuhata G. K., Smith M. W. (1981). History of health studies around nuclear facilities: a methodological consideration.. Environ Res.

[OCR_01060] Urquhart J., Palmer M., Cutler J. (1984). Cancer in Cumbria: the Windscale connection.. Lancet.

